# Oral treatments for fungal infections of the skin of the foot

**DOI:** 10.1590/1516-3180.20141322T1

**Published:** 2014-04-01

**Authors:** Sally E. M. Bell-Syer, Sameena M. Khan, David J. Torgerson

## Abstract

**BACKGROUND:**

About 15% of the world population have fungal infections of the feet (tinea pedis or athlete's foot). There are many clinical presentations of tinea pedis, and most commonly, tinea pedis is seen between the toes (interdigital) and on the soles, heels, and sides of the foot (plantar). Plantar tinea pedis is known as moccasin foot. Once acquired, the infection can spread to other sites including the nails, which can be a source of re-infection. Oral therapy is usually used for chronic conditions or when topical treatment has failed.

**OBJECTIVE:**

To assess the effects of oral treatments for fungal infections of the skin of the foot (tinea pedis).

**METHODS:**

*Search methods:* For this update we searched the following databases to July 2012: the Cochrane Skin Group Specialized Register, CENTRAL in The Cochrane Library, MEDLINE (from 1946), EMBASE (from 1974), and CINAHL (from 1981). We checked the bibliographies of retrieved trials for further references to relevant trials, and we searched online trials registers. *Selection criteria:* Randomized controlled trials of oral treatments in participants who have a clinically diagnosed tinea pedis, confirmed by microscopy and growth of dermatophytes (fungi) in culture. *Data collection and analysis:* Two review authors independently undertook study selection, "Risk of bias" assessment, and data extraction.

**MAIN RESULTS:**

We included 15 trials, involving 1,438 participants. The 2 trials (71 participants) comparing terbinafine and griseofulvin produced a pooled risk ratio (RR) of 2.26 (95% confidence interval (CI) 1.49 to 3.44) in favors of terbinafine's ability to cure infection. No significant difference was detected between terbinafine and itraconazole, fluconazole and itraconazole, fluconazole and ketoconazole, or between griseofulvin and ketoconazole, although the trials were generally small. Two trials showed that terbinafine and itraconazole were effective compared with placebo: terbinafine (31 participants, RR 24.54, 95%CI 1.57 to 384.32) and itraconazole (72 participants, RR 6.67, 95%CI 2.17 to 20.48). All drugs reported adverse effects, with gastrointestinal effects most commonly reported. Ten of the trials were published over 15 years ago, and this is reflected by the poor reporting of information from which to make a clear "Risk of bias" assessment. Only one trial was at low risk of bias overall. The majority of the remaining trials were judged as "unclear" risk of bias because of the lack of clear statements with respect to methods of generating the randomization sequence and allocation concealment. More trials achieved blinding of participants and personnel than blinding of the outcome assessors, which was again poorly reported.

**AUTHORS' CONCLUSIONS:**

The evidence suggests that terbinafine is more effective than griseofulvin; and terbinafine and itraconazole are more effective than no treatment. In order to produce more reliable data , a rigorous evaluation of different drug therapies needs to be undertaken with larger sample sizes to ensure they are large enough to show any real difference when two treatments are being compared. It is also important to continue to follow up and collect data , preferably for six months after the end of the intervention period, to establish whether or not the infection recurred.

**This is the abstract of a Cochrane Review published in the Cochrane Database of Systematic Reviews (CDSR) 2012, issue 10, Art. No. CD003584. DOI:** 10.1002/14651858.CD003584.pub2 (http://onlinelibrary.wiley.com/doi/10.1002/14651858.CD003584.pub2/full). For full citation and authors details see reference 1.

**The full text is available from:**http://onlinelibrary.wiley.com/store/10.1002/14651858.CD003584.pub2/asset/CD003584.pdf?v=1&t=hralbcjp&s=8a122343e91a325aeb541587596a057767c41698

The abstract is also available in the Portuguese, Spanish and Chinese languages from: http://summaries.cochrane.org/CD003584/oral-antifungal-drugs-for-treating-athletes-foot-tinea-pedis#sthash.i0KoYuHh.dpuf

## COMMENTS

*"Tinea pedis"*, which includes interdigital mycosis (i.e. between the toes) and onychomycosis (nail mycosis), is a very important and frequent problem and needs to be well treated. Nail mycosis can be a source of reinfection. Itraconazole is the most effective agent since it eliminates dermatophytes, yeasts and also filamentous fungi, while terbinafine is effective against dermatophytes.

Cracks, scaling or maceration in the toe-web spaces (interdigital mycosis) may be the sites where beta-hemolytic streptococci enter to cause cellulitis of the legs. However, these sites are often little heeded. In 85% of a group of patients with cellulitis and tinea pedis, cultures yielded beta-hemolytic streptococci (Lancefield group A in 4, group B in 3, group C in 1 and group G in 9). In contrast, in a control group of 30 patients with tinea pedis but without cellulitis, beta-hemolytic streptococci were not isolated from the interdigital spaces. The growth of Gram-negative bacilli and *Staphylococcus aureus* was similar in the two patient populations.^1^ The bacteria may cause cellulitis by entering the skin at these sites or by spreading to contiguous cutaneous surfaces and invading through a disrupted epidermis or through areas of impaired local defenses.

This correlation aims to emphasize the importance of seeking and treating tinea pedis, so as to prevent recurrent episodes of cellulitis, which are common in patients who have had a previous attack. 

**Leontina da Conceição Margarido**, MD, PhD. Professor at the Biological and Health Sciences Center, Mackenzie Presbyterian University; Chairman of the Department of Dermatology, Associação Paulista de Medicina (APM); Counselor of the Brazilian Society of Dermatology.
